# Case report: *De novo SAMD9L* truncation causes neonatal-onset autoinflammatory syndrome which was successfully treated with hematopoietic stem cell transplantation

**DOI:** 10.3389/fped.2023.1108207

**Published:** 2023-03-10

**Authors:** María Soledad Caldirola, Analía Gisela Seminario, Paula Carolina Luna, Renata Curciarello, Guillermo Horacio Docena, Nicolás Fernandez Escobar, Guillermo Drelichman, Marco Gattorno, Adriana A. de Jesus, Raphaela Goldbach-Mansky, María Isabel Gaillard, Liliana Bezrodnik

**Affiliations:** ^1^ Servicio de Inmunología, “Hospital de Niños “Dr. Ricardo Gutiérrez,” Buenos Aires, Argentina; ^2^ Instituto Multidisciplinario de Investigaciones en Patologías Pediátricas (IMIPP-CONICET-GCBA), Buenos Aires, Argentina; ^3^ Centro de Inmunología Clínica Dra. Bezrodnik y equipo, Buenos Aires, Argentina; ^4^ Servicio de Dermatología, Hospital Alemán, Buenos Aires, Argentina; ^5^ Instituto de Estudios Inmunológicos y Fisiopatológicos (IIFP)-CONICET-UNLP, Dto. de Cs Biológicas, Facultad de Ciencias Exactas, La Plata, Buenos Aires, Argentina; ^6^ Unidad de Trasplante de Médula Ósea-Fundación Favaloro, Buenos Aires, Argentina; ^7^ UOC Reumatologia e Malattie Autoinfiammatorie, IRCCS Istituto Giannina Gaslini, Genova, Italy; ^8^ Translational Autoinflammatory Diseases Section, NIAID/NIH, Bethesda, MD, United States; ^9^ Sección Citometría-Laboratorio Stamboulian, Buenos Aires, Argentina

**Keywords:** autoinflammatory syndromes, CANDLE-like syndrome, primary immunodeficiencies, SAMD9L, sterile alpha motif domain containing 9 like, case report

## Abstract

During recent years, the identification of monogenic mutations that cause sterile inflammation has expanded the spectrum of autoinflammatory diseases, clinical disorders characterized by uncontrolled systemic and organ-specific inflammation that, in some cases, can mirror infectious conditions. Early studies support the concept of innate immune dysregulation with a predominance of myeloid effector cell dysregulation, particularly neutrophils and macrophages, in causing tissue inflammation. However, recent discoveries have shown a complex overlap of features of autoinflammation and/or immunodeficiency contributing to severe disease phenotypes. Here, we describe the first Argentine patient with a newly described frameshift mutation in *SAMD9L* c.2666delT/p.F889Sfs*2 presenting with a complex phenotypic overlap of CANDLE-like features and severe infection-induced cytopenia and immunodeficiency. The patient underwent a fully matched unrelated HSCT and has since been in inflammatory remission 5 years post-HSCT.

## Introduction

During recent years, the identification of monogenic causes of systemic autoinflammatory diseases (SAIDs) uncovered the role of innate immune sensing linked to specific proinflammatory cytokines and their signaling pathways as a cause of sterile inflammation. The availability of genetic testing has allowed physicians all over the world to make specific diagnoses in an increasing number of patients with rare severe immune dysregulation diseases (1). The clinical hallmark of SAIDs is the presence of recurrent fevers and other systemic signs of inflammation with nonspecific elevation in acute reactant markers (2, 3). Moreover, SAIDs can be misdiagnosed with systemic infections and/or immunodeficiency due to severe uncontrolled systemic and organ-specific inflammation (4, 5). The first group of monogenic SAIDs presented with dysregulation in IL-1β production, including the IL-1β activating inflammasomopathies. A recently described group of SAIDs that present with chronic upregulation of type 1 interferon (IFN)-stimulated or -regulated genes (IRGs) is referred to as autoinflammatory interferonopathies (4, 6, 7).

However, recent discoveries of additional monogenic diseases, including a disease caused by truncating mutations in *SAMD9L*, illustrate the existence of a complex overlap of autoinflammatory manifestations, autoimmune diseases and/or immunodeficiencies that have been included in the latest Classification of the International Union of Immunological Societies of Inborn Errors of Immunity (1, 8–10). Among these, homozygous loss-of-function mutations in *PSMB8* (proteasome subunit β type 8) were first described to cause CANDLE syndrome (chronic atypical neutrophilic dermatosis with lipodystrophy and elevated temperature), a disease that presents with systemic inflammation, neutrophilic panniculitis, and a chronically elevated IRG signature (11). Later, mutations in different protein subunits of the proteasome–immunoproteasome system (12, 13) or proteasome assembly molecules (13, 14) were published. The prominent peripheral panniculitis in CANDLE syndrome that results in lipodystrophy is a hallmark feature that is however also seen in patients presenting with SAMD9L truncating mutations in *SAMD9L* and has been previously reported in patients with a wide spectrum of hematological diseases: ataxia-pancytopenia syndrome (ATXPC), aplastic anemia, cytopenia, myelodysplastic syndrome (MDS) predisposition (resembling monosomy 7 and interstitial deletion of 7q disorders), myeloid malignancies, infections, and immunodeficiency (15–19). In most instances, the disease presents later in life or even in adulthood. In 2020, de Jesus et al. published, for the first time, *de novo* heterozygous truncating *SAMD9L* mutations that cause a severe perinatal onset SAID (20). Here, we describe the first Argentine patient who presented with neutrophilic panniculitis and lipodystrophy in the context of systemic inflammation that mimicked neonatal-onset CANDLE-like autoinflammatory disease. We now report her long-term follow-up after a successful hematopoietic stem cell transplantation (HSCT). This report raises awareness of *SAMD9L* truncating mutations that cause severe cytopenias and autoinflammation and guides treatment decisions.

## Case presentation

**Figure 1:**
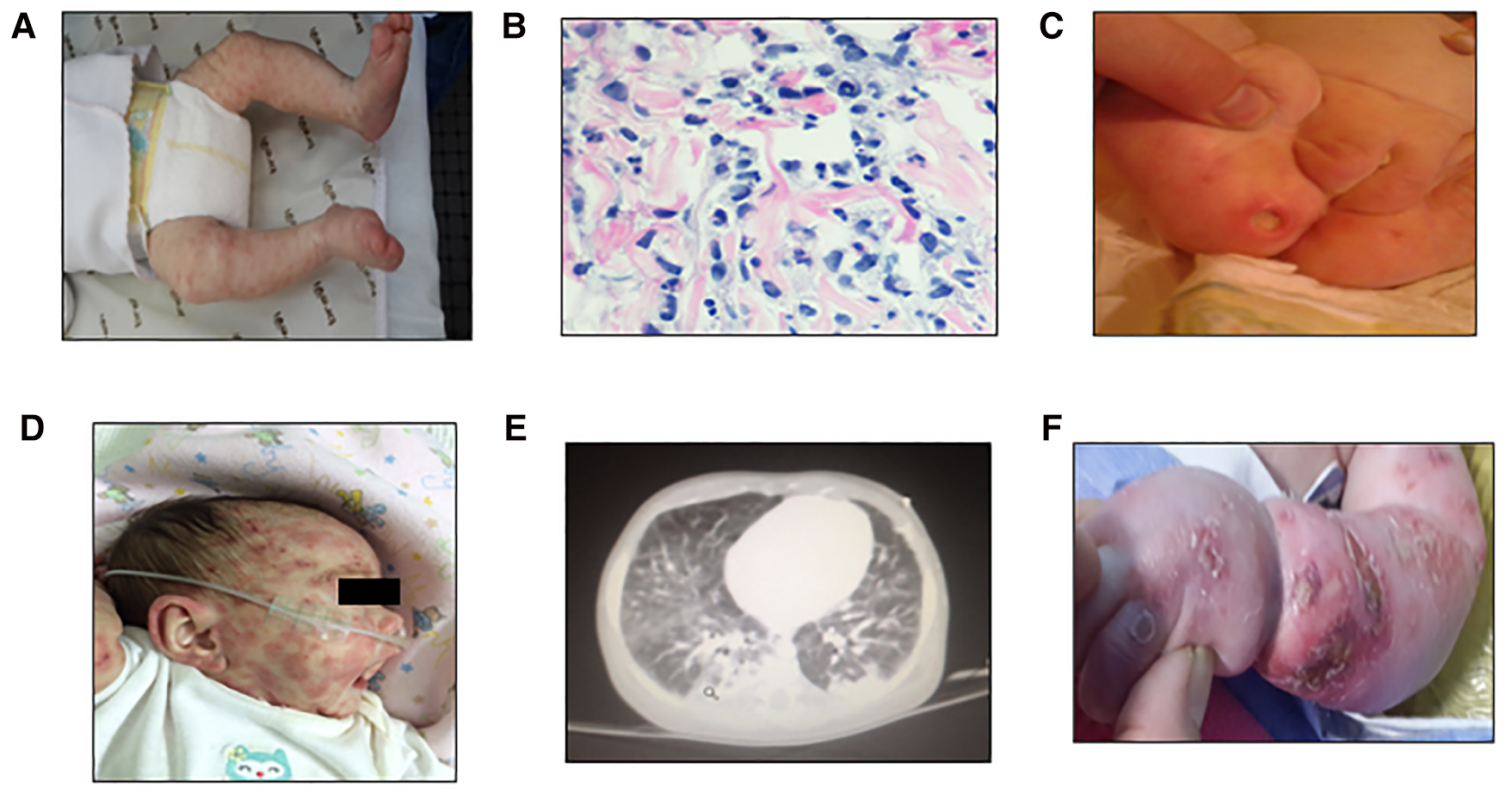
First skin lesions: nonevanescent erythematus papular generalized eruption at the first consult 24 days since birth (**A**), H&E staining showing interstitial infiltrates consisting of some atypical mononuclear cells with karyorrhexis (**B**), and persistent severe skin ulcers at 2 months of age (**C**), skin exacerbations previous to anti-TNFα therapy (**D**), lung MRI showing inflammatory infiltrate at 6 months after the fifth dose of anti-TNFα therapy (**E**), and skin erythema exacerbation in arms and legs prior to HSCT at 8 months (**F**).

A 5-year-old girl, the first child from nonconsanguineous parents, was born at 38 weeks with no remarkable familiar history. At 1 week of life, she developed a nonevanescent erythematous-papular generalized eruption without fever, visceromegaly, or joint involvement (Figure 1A). A skin biopsy and laboratory evaluation were performed. Her first laboratory evaluation at 24 days of life revealed an elevated white blood cell (WBC) count (32.6 × 10^9^/L) and elevated acute phase reactants: erythrocyte sedimentation rate (ESR) of 68 mm/h (normal <15 mm/h) and C-reactive protein (CRP) level of 78.5 mg/L (normal <5.0 mg/L). Her initial immunological studies showed hypogammaglobulinemia, extremely low B cells, normal T and NK cell counts with T lymphocyte subset frequencies, and proliferative response to mitogens within range values for age (see Table 1). The skin biopsy revealed interstitial infiltrates of some atypical mononuclear cells with karyorrhexis (Figure 1B). She started on IVIG (1 g/kg/21 days) and corticosteroids (1 mg/kg). At 2 months, the severe skin lesions persisted, and she developed superinfected ulcerations on her buttocks, anemia (Hb: 90 g/L), and splenomegaly (Figure 1C). At 3 months, ADA2 enzyme activity was found to be normal at Dr. Michael S. Hershfield's Lab in Duke University Medical Center. The analysis of the *IL1RN* gene excluded a possible interleukin-1 receptor antagonist (DIRA). A brain MRI was normal (no calcifications), and height and weight were below the third percentile; developmentally, she lost her social smile. Due to persistent exacerbations of skin lesions, prednisone at 2 mg/kg/day and antibiotics (levofloxacin, 10 mg/kg/12 h) were started without improvement (Figure 1D). At the age of 4 months, anti-TNFα therapy with adalimumab at 0.8 mg/dose every week was started. Her WBC counts normalized. Acute phase reactants dropped, and the skin lesions improved, but she developed progressive B and NK cell cytopenia. At 6 months, after the fifth dose of adalimumab, she developed new severe skin lesions, lymphopenia, anemia, thrombocytopenia, visceromegaly, and respiratory failure requiring mechanical ventilation; biologics were discontinued (Figure 1E). She developed skin erythema and adenopathy at the BCG site, and blood culture was positive for *S. aureus* (Figure 1F). As the patient had a new episode of cytopenia, we added cyclosporine to previous medications with a partial response. Other molecular studies showed no mutation in *CECR1/ADA2*, *MVK*, *TNFRSF1A*, *TMEM173/STING1*, *NLRP3*, *PSMB8*, *RAG1*, and *RAG2* genes. Due to her poor general condition, anti-IL1β treatment with anakinra at 1 mg/kg/dose was started, and cytokine studies revealed high levels of IL-6 and IL-8. The patient remained clinically stable with no major symptoms, but *Mycobacterium bovis* was isolated from the blood, which prompted tuberculostatic treatment with four drugs. Her lung disease improved, and she was extubated and remained clinically stable. Colleagues agreed with the diagnosis of SAID. Considering her previous and persistent clinical severity since birth, the decision to treat her with an HSCT was made.

**Table 1:** Laboratory findings before Hematopoietic stem cell transplantation.

	Patient (24 days old)	Patient (1 m)	Patient (4 m)	Patient (6 m)
Hemoglobin (g/dl)	13.0	**9.0**	**9.5**	**8.5**
WBC (10^3^/µl)	**32.6**	**21.0**	5.4	8.0
Banded neutrophils (%)	8	6	0	0
Neutrophils (%)	61	57	40	80
Eosinophils (%)	4	3	2	3
Basophils (%)	0	0	0	0
Lymphocytes (%)	19	19	38	9
Monocytes (%)	8	16	20	8
Platelets (10^3^/µl)	182	224	160	**31.2**
CRP (mg/L)	**20.0**	**78.5**	**20.4**	ND
ERS (mm/h)	**40**	**68**	**40**	ND
C3 (mg/dl)	196	ND	ND	ND
C4 (mg/dl)	26	ND	ND	ND
IgG (mg/dl)	**424**	**409**	1,049^*^	1,285^*^
IgA (mg/dl)	**<15**	**<15**	38	93
IgM (mg/dl)	**9**	**8**	10	39
IgE (UI/ml)	5.6	ND	ND	ND
CD3% (mm^3^)	69 (3,827)	74 (2,953)	96 (1,919)	**94 (602)**
CD4% (mm^3^)	45 (2,496)	48 (1,915)	63 (1,259)	**60 (384)**
CD8% (mm^3^)	24 (1,331)	26 (1,037)	32 (639)	**34 (218)**
CD19% (mm^3^)	**2 (111)**	**2 (80)**	**0.2 (4)**	**0.23 (1.4)**
CD56% (mm^3^)	24 (1,331)	22 (878)	**4 (80)**	**2 (13)**
TCR αβ %	99.5	ND	ND	ND
TCR γδ %	0.5	ND	ND	ND
DN TCR αβ CD3^+^ cells %	0.3	ND	ND	ND
Naϊve CD4^+^ %	86	ND	ND	ND
Central memory CD4^+^ %	13.7	ND	ND	ND
Effector memory CD4^+^ %	0.3	ND	ND	ND
Terminal effector CD4^+^ %	0.0	ND	ND	ND
HLA-DR CD4^+^ %	2.7	ND	ND	ND
Naϊve CD8^+^ %	93	ND	ND	ND
Central memory CD8^+^ %	3.3	ND	ND	ND
Effector memory CD8^+^ %	0.7	ND	ND	ND
Terminal effector CD8^+^ %	3.0	ND	ND	ND
HLA-DR CD8^+^ %	2.0	ND	ND	ND
Naϊve CD19^+^ %	NA	NA	85	ND
IgM memory B cells %	NA	NA	0.25	ND
Switched memory %	NA	NA	14	ND
Transitional B cells %	NA	NA	2	ND
CD21^low^ %	NA	NA	ND	ND
Plasmablasts %	NA	NA	ND	ND

* Under intravenous immunoglobulin treatment.

At 10 months of age, the patient received an unrelated-donor hematopoietic stem cell transplantation, fully matched, MHC compatibility degree (10/10), with no molecular diagnosis. The conditioning regimen consisted of busulphan (4.8 mg/kg/dose IV for 4 days), fludarabine (40 mg/m^2^/dose IV for 4 days), and thymoglobulin (2.25 mg/kg/dose IV for 2 days). GVHD prophylaxis with tacrolimus and methotrexate (with leucovorin rescue) was administered. The source of stem cells was bone marrow (dose: 7 × 10^6^ CD34 cells/kg/body weight). Neutrophil engraftment was achieved on day +17, and platelet engraftment was achieved on day +18. She did not present graft vs. host disease, and chimerism was evaluated by PCR, resulting in 100% donor cells. She was discharged from the clinic on day +47. A month later, Sanger sequencing trio performed at the NIAID/NIH revealed a *de novo*, heterozygous variant in *SAMD9L* c.2666delT/p.F889Sfs*2 with normal IFN-response-gene score (20).

**Figure 2:**
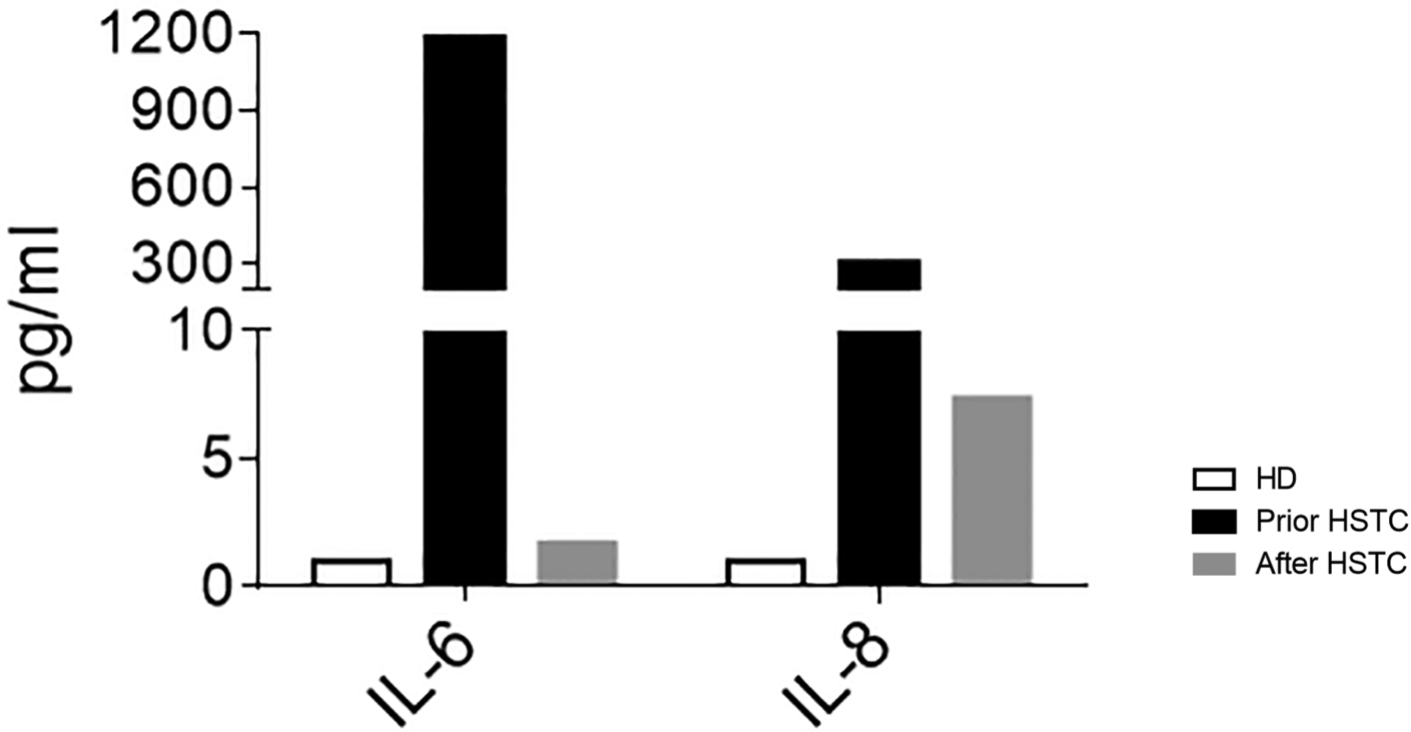
Plasma levels of IL-6 and IL-8 in HD (*n* = 5) and the patient before HSCT (black bars) and on day +420 after HSCT (gray bars).

At 6 months after the transplant, patient's WBC, CRP, and ERS were within reference values and she had recovered the B cell count (32% and 624/mm^3^, respectively). Between 12 and 14 months, we evaluated T and B lymphocyte compartments, which revealed beginning T cell reconstitution. We further confirmed a marked decrease in serum IL-6 and IL-8 levels in peripheral blood (Figure 2); 2 years after HSCT, the girl has fully acquired immune reconstitution, showing memory B cells and normal CD4^+^ and CD8^+^ proliferations to PHA, OKT3, and SEB (see Table 2). Her tracheotomy could not be removed on two different opportunities (2 and 4 years old) due to mucosal fibrosis and her low weight. It is worth mentioning that during an influenza episode and a second nasopharyngeal probe placement, she presented with a severe mucosal inflammation exacerbation episode requiring mechanical ventilation in the ICU that resolved with corticosteroid treatment. At present, 5 years post-HSCT, she has mild cognitive retardation, she still uses diapers, and her speech is difficult to evaluate as she still has tracheotomy. Moreover, height and weight remain below the third percentile for age. During the COVID pandemic, she had an adenovirus infection that required oxygen and steroids. Her left eye is blind due to retinal detachment. She achieved full donor chimerism without skin lesions, has recovered peripheral blood B cells with immune reconstitution, and is otherwise in good health (see Table 2).

**Table 2:** Laboratory findings after hematopoietic stem cell transplantation.

	Patient (1 m)	Patient (6 m)	Patient (12 m)	Patient (14 m)	Patient (26 m)	Patient (35 m)	Patient (67 m)
Hemoglobin (g/dl)	NA	8.7	12.7	13.4	13.0	12.2	12.9
WBC (10^3^/µl)	**18.6**	4.1	4.8	4.7	5.3	7.3	9.7
Banded neutrophils (%)	NA	0	2	0	0	0	0
Neutrophils (%)	NA	43	29	33	39	42	63
Eosinophils (%)	NA	2	3	3	1	3	2
Basophils (%)	NA	0	1	0	0	0	0
Lymphocytes (%)	8	48	55	52	52	47	26
Monocytes (%)	NA	7	10	12	8	8	9
Platelets (10^3^/µl)	NA	60	192	201	273	325	398
CRP (mg/L)	ND	ND	1.8	0.7	ND	0.5	**7.5**
ERS (mm/h)	ND	14.5	**60**	**26**	ND	**19**	**33**
C3 (mg/dl)	ND	ND	ND	ND	120	116	159
C4 (mg/dl)	ND	ND	ND	ND	21	23	30
IgG (mg/dl)	ND	1,043^a^	1,064^a^	1,134^a^	1,061^a^	1,053^a^	961^a^
IgA (mg/dl)	ND	54	82	66	80	97	215
IgM (mg/dl)	ND	42	124	84	68	95	105
IgE (UI/ml)	ND	3.8	<1.5	1.9	<1.5	2.7	2.1
CD3% (mm^3^)	91 (1,354)	59 (1,150)	79 (2,085)	74 (1,797)	70 (1,933)	69 (2,351)	66 (1,665)
CD4% (mm^3^)	11 (164)	19 (370)	22 (581)	20 (486)	21 (580)	21 (718)	26 (656)
CD8% (mm^3^)	78 (1,161)	36 (702)	46 (1,214)	49 (1,190)	41 (1,132)	41 (1,392)	36 (908)
CD19% (mm^3^)	0 (0)	32 (624)	13 (343)	18 (437)	21 (580)	17.8 (608)	14.6 (368)
CD56% (mm^3^)	9 (134)	6 (117)	8 (211)	7 (170)	5.9 (163)	11 (375)	15.9 (401)
TCR αβ %	ND	ND	87	88	87	ND	ND
TCR γδ %	ND	ND	13	12	13	ND	ND
DN CD3^+^ cells %	ND	ND	0.9	ND	1.4	ND	ND
Naϊve CD4^+^ %	3	ND	9.6	12.7	25.7	ND	31.8
Naϊve CD4^+^ RTE %	66	ND	99	93	78	ND	75
Central memory CD4^+^ %	50.6	ND	36.6	34.5	43.2	ND	46.1
Effector memory CD4^+^ %	42	ND	50.1	49.6	30.0	ND	22.1
Terminal effector CD4^+^ %	4.4	ND	3.7	3.2	1.1	ND	0.1
HLA-DR CD4^+^ %	38	ND	54	54	37	ND	29.1
Naϊve CD8^+^ %	11.6	ND	7.7	7.4	14.4	ND	17.8
Central memory CD8^+^ %	28.7	ND	24.2	25.0	27.2	ND	35.8
Effector memory CD8^+^ %	40.8	ND	32.6	33.9	20.0	ND	38.3
Terminal effector CD8^+^ %	18.9	ND	35.5	33.6	38.4	ND	8.1
HLA-DR CD8^+^ %	89	ND	68	74	78	ND	69.4
Naϊve CD19^+^ %	NA	NA	83	82	72.4	ND	53.7
IgM memory B cells %	NA	NA	3.0	4.8	8.0	ND	12.7
Switched memory %	NA	NA	4.5	5.6	10.3	ND	17.2
Transitional B cells %	NA	NA	13	10	3.2	ND	7.0
CD21^low^ %	NA	NA	3.0	3.4	2.1	ND	5.0
Plasmablasts %	NA	NA	4.9	3	3.3	ND	**6.4**

Bold numbers represent abnormal values for age-matched donors.

^a^ Under IVIG treatment; ND, not done; NA, not applicable. T cells (gated on CD3^+^ CD4^+^ or CD3^+^CD8^+^ cells): naïve T cells (CD45RA^+^CD27^+^); recent thymic emigrants (naïve CD4+ RTE, gated on CD45RA^+^CD27^+^); central memory T cells (CD45RA^−^CD27^+^); effector memory T cells (CD45RA^−^CD27^−^); terminal effector T cells (CD45RA^+^CD27^−^); B cells (gated on CD19^+^ cells): naïve B cells (IgD^+^CD27^−^); IgM memory B cells (IgM^+^IgD^−^ CD27^+^); switched memory (IgD^−^IgM^−^CD38^+/−^); transitional B cells (CD38^++^CD24^++^); CD21^low^ B cells (CD21^low^CD38^+/−^); plasmablasts (CD38^++^CD27^++^).

## Discussion

Monogenic AD is an evolving group of complex inborn errors of immunity. In many instances, the clinical presentation of cytopenias, immunodeficiencies, and autoinflammation can be confusing, and the inability to control systemic inflammation can result in poor outcomes (21). In this report, we describe a challenging patient who presented with neonatal-onset CANDLE-like features diagnosed with a *de novo* truncating *SAMD9L* mutation and who was successfully treated with an HSCT.

In recent years, germline heterozygous missense mutations in *SAMD9L* have been reported as the monogenic causes for sporadic or autosomal dominant cases of ATXPC and MDS (15, 17, 19, 22), but these patients do not present with early-onset disease and their inflammatory markers are normal. These clinical presentations differed from the early onset severe manifestations described by de Jesus et al., who reported a cohort of SAID patients in which some had monogenic truncating mutations in SAMD9L that presented with IFN and NFκB-mediated dysregulation during flares (20). In total, eight patients with SAMD9L-SAAD have been described so far: three are deceased (one in the context of a BMT), and two have been successfully bone marrow-transplanted including this patient. Severe and progressive B and NK cell cytopenias without alterations of myeloid subpopulations have also been described (22).

Patients with missense *SAMD9L* mutations who developed MDS have received HSCT, resulting in high donor engraftment (23). We now report that HSCT also leads to the resolution of the systemic and skin manifestations 5 years post-transplantion and provides a positive outcome on an HSCT that was conducted in a life-threatening disease with no good treatment options. However, questions regarding the long-term management of transplanted patients remain unanswered, including the safety of vaccinations, continued benefit to skin inflammation and viral infections, and the prevention of the development of cerebellar atrophy, a feature seen in adults with missense mutations.

In summary, this report raises awareness of SAMD9L-SAAD as a differential diagnosis of a CANDLE-like syndrome with immunodeficiency, and our data suggest HSCT as a treatment option.

## Data Availability

The original contributions presented in the study are included in the article/supplementary materials; further inquiries can be directed to the corresponding author.
